# T Cell Clonal Analysis Using Single-cell RNA Sequencing and Reference Maps

**DOI:** 10.21769/BioProtoc.4735

**Published:** 2023-08-20

**Authors:** Massimo Andreatta, Paul Gueguen, Nicholas Borcherding, Santiago J. Carmona

**Affiliations:** 1 Ludwig Institute for Cancer Research, Lausanne Branch, and Department of Oncology, CHUV and University of Lausanne, Epalinges, Switzerland; 2 Agora Cancer Research Center, Lausanne, Switzerland; 3 Swiss Institute of Bioinformatics, Lausanne, Switzerland; 4 Department of Pathology & Immunology, Washington University in St. Louis, St. Louis, MO, USA

**Keywords:** Single-cell analysis, T-cell receptor, TCR, Transcriptomics, T-cell clone, Reference projection, scRNA-seq, scTCR-seq

## Abstract

T cells are endowed with T-cell antigen receptors (TCR) that give them the capacity to recognize specific antigens and mount antigen-specific adaptive immune responses. Because TCR sequences are distinct in each naïve T cell, they serve as molecular barcodes to track T cells with clonal relatedness and shared antigen specificity through proliferation, differentiation, and migration. Single-cell RNA sequencing provides coupled information of TCR sequence and transcriptional state in individual cells, enabling T-cell clonotype-specific analyses. In this protocol, we outline a computational workflow to perform T-cell states and clonal analysis from scRNA-seq data based on the R packages Seurat, ProjecTILs, and scRepertoire. Given a scRNA-seq T-cell dataset with TCR sequence information, cell states are automatically annotated by reference projection using the ProjecTILs method. TCR information is used to track individual clonotypes, assess their clonal expansion, proliferation rates, bias towards specific differentiation states, and the clonal overlap between T-cell subtypes. We provide fully reproducible R code to conduct these analyses and generate useful visualizations that can be adapted for the needs of the protocol user.

Key features

Computational analysis of paired scRNA-seq and scTCR-seq data

Characterizing T-cell functional state by reference-based analysis using ProjecTILs

Exploring T-cell clonal structure using scRepertoire

Linking T-cell clonality to transcriptomic state to study relationships between clonal expansion and functional phenotype

Graphical overview

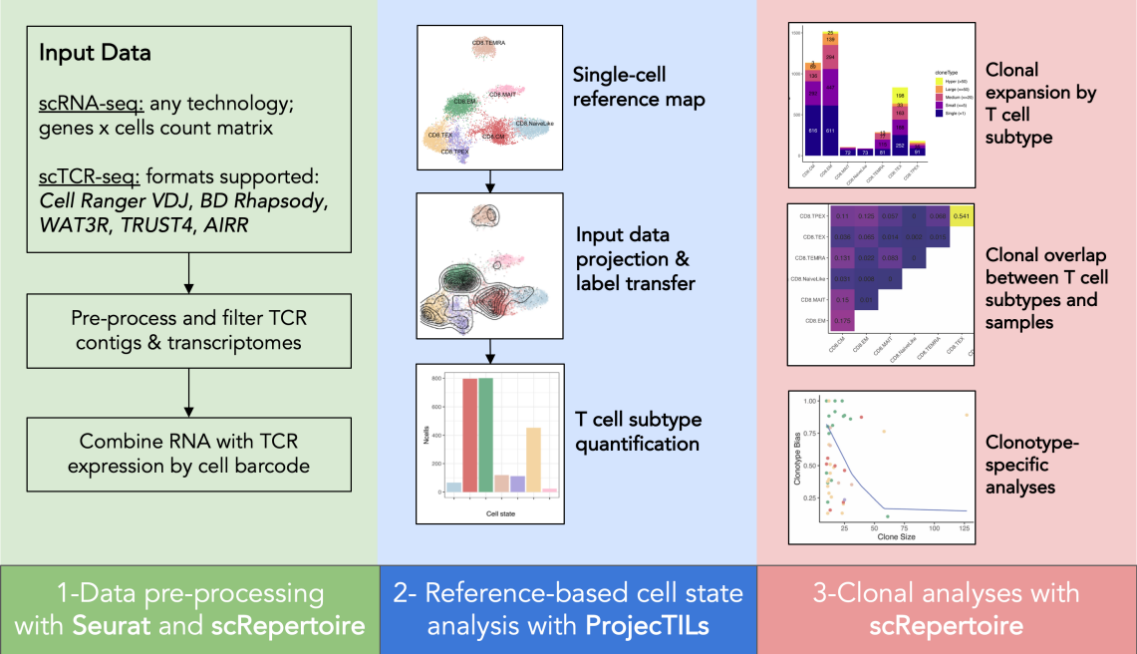

## Background

T cells are crucial players in the adaptive immune response with the capacity to recognize and eliminate infected and malignant cells. The antigen specificity of T cells is conferred by their T-cell receptors (TCRs). These are heterodimeric proteins in which each of the two protein chains—typically one alpha (α) and one beta (β)—is produced through somatic rearrangement of V, (D), and J gene segments, as well as the addition or deletion of nucleotides between spliced gene segments, to form a unique V(D)J exon. This recombination process is largely random and generates a large repertoire of TCRs, with an estimated diversity in the order of 10^8^–10^10^ unique TCR chains in a single individual (Qi et al., 2014; Lythe et al., 2016). Such a large repertoire, and in particular the hypervariable complementary-determining region 3 (CDR3) of the TCR, allows recognizing with high specificity a vast array of foreign antigens, while maintaining tolerance to self-antigens. Due to this huge diversity, each naïve αβ T cell has a virtually unique combination of TCR α and β chains. Thus, paired αβ TCR sequences serve as molecular barcodes to track T-cell clones through processes such as proliferation, differentiation, and migration.

The emergence of single-cell technologies has enabled the coupled sequencing of full-length TCRs with transcriptome-wide RNA sequencing in individual cells (Pai and Satpathy, 2021). This is achieved by either computationally reconstructing TCR chain transcript sequences from single-cell RNA sequencing reads (Eltahla et al., 2016; Stubbington et al., 2016; Bolotin et al., 2017) or by specifically amplifying the TCR locus, also known as single-cell TCR-seq (e.g., using Chromium 5′ V(D)J sequencing). Coupled with scTCR-seq, scRNA-seq enables the connection between T-cell states, clonotypes, and potential antigen specificity (Han et al., 2014).

We have recently proposed computational pipelines for the analysis of single-cell T-cell repertoires (Borcherding and Bormann, 2020) and for reference-based analysis of single-cell transcriptomics data (Andreatta et al., 2021a), based on the tools scRepertoire and ProjecTILs, respectively. In this protocol, we describe how to combine these computational tools to analyze paired scRNA-seq and scTCR-seq data to track individual clonotypes projected in a reference map, assess their clonal expansion, proliferation rates, their bias towards specific differentiation states, and the clonal overlap between T-cell states. We will focus the examples on human CD8^+^ T cells from tumor biopsies, but the protocol is applicable to any single-cell transcriptomics data with TCR sequence information in humans and mice. We invite the reader to follow this protocol while interactively running the associated R Notebook (see Software and datasets section).

## Equipment

1. Personal computer (minimum 16 GB of RAM) or high-performance computing cluster. All software runs on Linux, Windows, or MacOS machines.

## Software and datasets

This protocol requires basic R programming skills: installing packages, running an R notebook, and adapting the code to the needs of the user.

All software used for this protocol is free and open source.

1. R version 4.2 or higher
2. scRepertoire (version ≥ 1.7) (Borcherding and Bormann, 2020) (https://github.com/ncborcherding/scRepertoire)
3. ProjecTILs (version ≥ 3.0) (Andreatta et al., 2021a) (https://github.com/carmonalab/ProjecTILs)
4. Seurat (version ≥ 4.3) (Hao et al., 2021) (https://github.com/satijalab/seurat)
  In addition, it is recommended to install R Studio Desktop (https://posit.co/downloads/) to interactively run the R Notebook that reproduces the results of this protocol (https://github.com/carmonalab/Tcell_clonal_analysis).
  To download the repository to your machine, run the following from command line:
  git clone git@github.com:carmonalab/Tcell_clonal_analysis.git
  Then, move to the newly created directory and open the project file (with .Rproj extension). Open the protocol notebook (protocol_CD8TIL_clonalAnalysis.Rmd) in R Studio and execute all commands in order. Note that the R Notebook makes use of the *renv* package (https://rstudio.github.io/renv/articles/renv.html) for straightforward installation of all required packages with the correct version and to ensure reproducibility of the results shown in this protocol.
  The protocol assumes the user has generated a single-cell transcriptomics dataset with TCR sequencing information for the same T cells or a subset thereof. There is no restriction on the sequencing technology used, if it generates i) a count matrix quantifying gene expression in single cells; and ii) TCR sequences, for paired αβ chains or single chains, with barcodes that can be mapped to transcriptomics measurements of the same cells.

## Procedure

The protocol details all steps required to go from scRNA-seq and scTCR-seq count matrices to T-cell clonal analysis in the context of a T-cell reference map. Each step includes example code snippets that highlight the R commands that accomplish the step. For the complete list of R commands that reproduce the results of this protocol, refer to the accompanying R Notebook (see Software and datasets section).

- **Single-cell data pre-processing**
  1. scRNA-seq data
    Several protocols and technologies are available for transcriptomics quantification using scRNA-seq. Sequencing protocols differ in terms of library preparation, read alignment to a reference genome, and quantification of transcripts, as reviewed in multiple publications (Vieth et al., 2019; Mereu et al., 2020). Sequencing facilities commonly offer read mapping and gene expression quantification to obtain a raw count expression matrix (for instance, using the Cell Ranger pipeline from 10× Genomics). From a raw counts matrix, generate a Seurat (Hao et al., 2021) object to store the counts:
    seurat <- CreateSeuratObject(counts = matrix)
    Note that Seurat also implements functions to load data from specific technologies, for example the *Read10X()* function to read count matrices from the popular 10× sequencing platform (see https://satijalab.org/seurat/reference/read10x).
  2. scTCR-seq data
    Obtaining single-cell TCR sequences requires specific protocols for amplification and sequencing of the V(D)J locus, or their reconstruction from whole-transcriptome sequencing. For an overview of scTCR-seq sequencing approaches, see the comprehensive review by Pai and Satpathy (2021).
    We assume that the user has performed V(D)J sequences assembly and clonotype calling. For 10× Chromium 5′ V(D)J libraries, such annotated V(D)J sequences (“contigs”) are obtained from FASTQ files using the Cell Ranger V(D)J pipeline (https://support.10xgenomics.com/single-cell-vdj/software/pipelines/latest/using/vdj). scRepertoire (Borcherding and Bormann, 2020) implements useful functions to process the V(D)J contigs annotation files generated by Cell Ranger. These files (usually named all_contig_annotations.csv for total, or filtered_contig_annotations.csv for high-confidence filtered contigs) contain detailed information for each V(D)J contig, including its cell barcode, length, V-D-J-C segments, the number of reads and distinct UMIs aligned to the contig, and a clonotype ID to which the contig was assigned.
    Load TCR α and β chains from Cell Ranger output files and combine them by cell barcode, using function *combineTCR()* from scRepertoire:
    S1 <- read.csv("Sample1/outs/filtered_contig_annotations.csv")
    S2 <- read.csv("Sample2/outs/filtered_contig_annotations.csv")
    contig_list <- list(S1, S2)
    combined <- combineTCR(contig_list, cells ="T-AB")
    For V(D)J contigs generated using different pipelines, please see the *loadContigs()* function from scRepertoire, which allows data pre-processing for multiple formats including TRUST4, BD Rhapsody, WAT3R, and AIRR.
*Note 1: It is often useful for further processing steps to generate keys for unique clonotype–sample combinations. As it may occur by chance that the same clonotype is observed in different individuals, these keys will allow discriminating between T cells with identical TCR but from different samples. For example, generate a clonotype–sample key as a metadata column named “cdr3s_pat”:*
*combined <- lapply(combined, function(x){x$cdr3s_pat <- paste(x$CTaa, x$sample, sep="_"); x})*
  3. Combine scRNA-seq and scTCR-seq data
    Append the TCR information into the previously prepared Seurat object that stores the scRNA-seq counts. If the V(D)J data were processed using *combineTCR()* from scRepertoire, you may apply the *combineExpression()* function:
    seurat <- combineExpression(combined, seurat,
    group.by = "sample",
    cloneTypes=c(Single=1, Small=5, Medium=10, Large=20, Hyperexpanded=50))
    For V(D)J data pre-processed using different pipelines, add the TCR chains as metadata to the Seurat object:
    seurat <- AddMetaData(seurat, tcr.chains)
- **Reference-based analysis**
  1. Load reference map
    Several reference single-cell maps for reference-based analysis are available from the ProjecTILs repository (https://github.com/carmonalab/ProjecTILs) and from SPICA (Andreatta et al., 2021a) (https://spica.unil.ch). For example, to analyze human CD8^+^ T cells, download and load the corresponding map (Figure 1):
    ref.file <- "CD8T_human_ref_v1.rds"
    download.file("https://figshare.com/ndownloader/files/38921366", destfile = ref.file)
    ref.cd8 <- load.reference.map("CD8T_human_ref_v1.rds")
    DimPlot(ref.cd8, cols = ref.cd8@misc$atlas.palette)
    **Figure 1.**
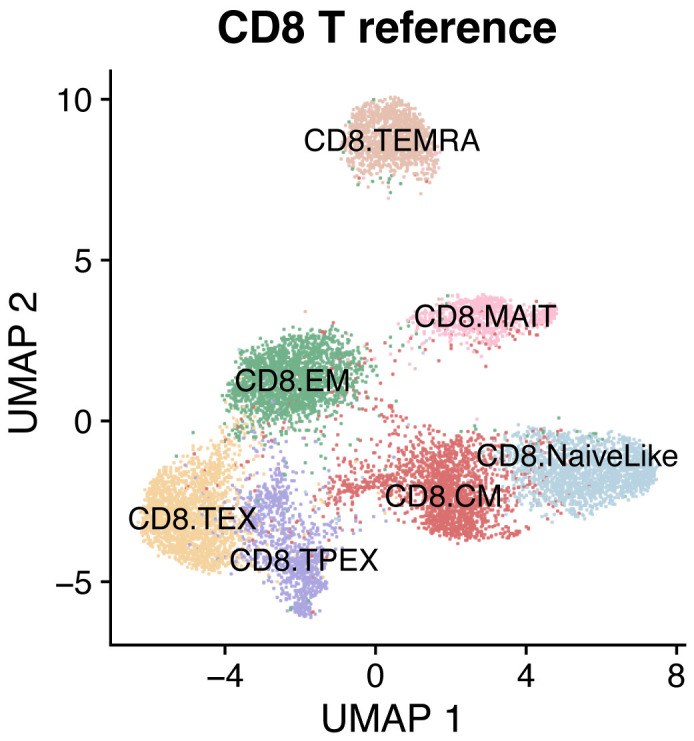
Reference map for human CD8^+^ T cells
  2. Project data into the reference
    To embed query data into the reference space and obtain cell type annotations, apply the ProjecTILs pipeline (Andreatta et al., 2021a). If the query dataset is composed of different samples (e.g., from different patients or time points), we recommend splitting it and projecting each sample separately into the reference. In this way, ProjecTILs will assume that each sample represents a different experimental batch and will calculate and correct batch effects accordingly:
    seurat.list <- SplitObject(seurat, split.by = "patient")
    seurat.projected <- Run.ProjecTILs(seurat.list, ref.cd8)
    In this case, the output is a list of Seurat objects, each corresponding to a query sample projected in the reference map.
    *Note 2: For this example, because we chose to use a CD8^+^ T cell reference map, ProjecTILs will automatically pre-filter CD8^+^ T cells from the input data (i.e., will remove CD4^+^ T cells and non-T cells). With ProjecTILs, it is also possible to conduct multi-reference map analysis, for instance using both CD8^+^ T cells and CD4^+^ T cells reference maps. An example can be found in the following R notebook:* *https://carmonalab.github.io/ProjecTILs_CaseStudies/Bassez_BC.html*.
  3. Compare marker gene expression profiles of query data with the reference map
    To verify the correspondence of transcriptional phenotypes between the reference and query dataset, visualize the average expression profile of each cell subtype for a panel of marker genes (Figure 2):
    which.patient <- "su009"
    plot.states.radar(ref.cd8, seurat.projected[[which.patient]], genes4radar = genes4radar)
    **Figure 2.**
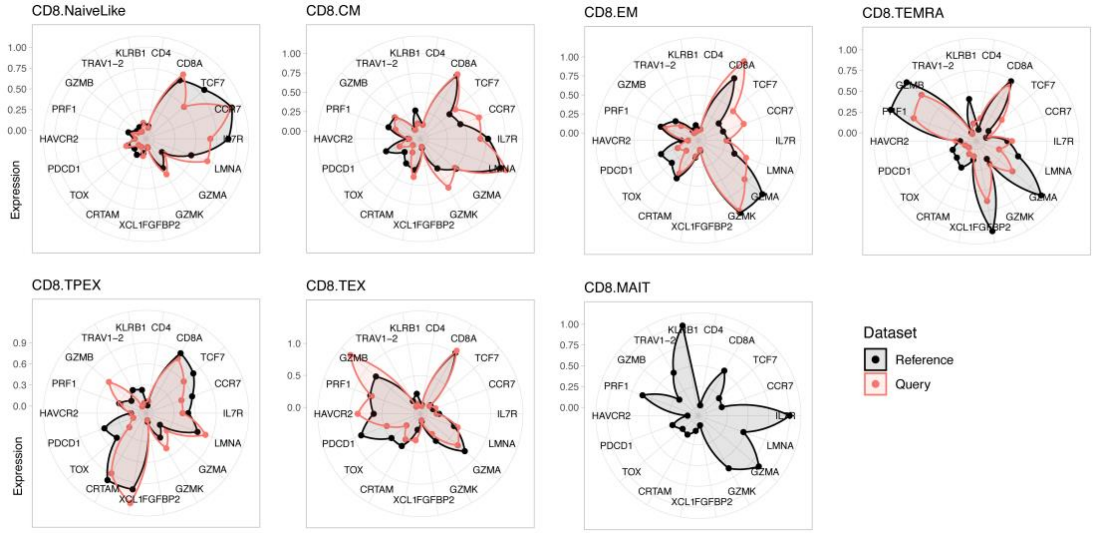
Expression profiles for reference and query dataset for a panel of marker genes. Reference is a human CD8^+^ T-cell reference (see text); the query is a representative individual (“su009”) from a cohort of basal cell carcinoma (BCC) patients (Yost et al., 2019).
  4. Cell subtype composition of query data
    Reference projection of the query data allows embedding them into the same space of the reference. Cell types for the query dataset can be predicted by nearest-neighbor majority voting based on the annotated reference cells. Visualize low-dimensional embeddings and subtype composition for individual samples or other subsets of the projected data (Figure 3):
    which.patient <- "su009"
    a <- plot.projection(ref.cd8, seurat.projected[[which.patient]])
    b <- plot.statepred.composition(ref.cd8, query = seurat.projected[[which.patient]])
    a | b
    **Figure 3.**
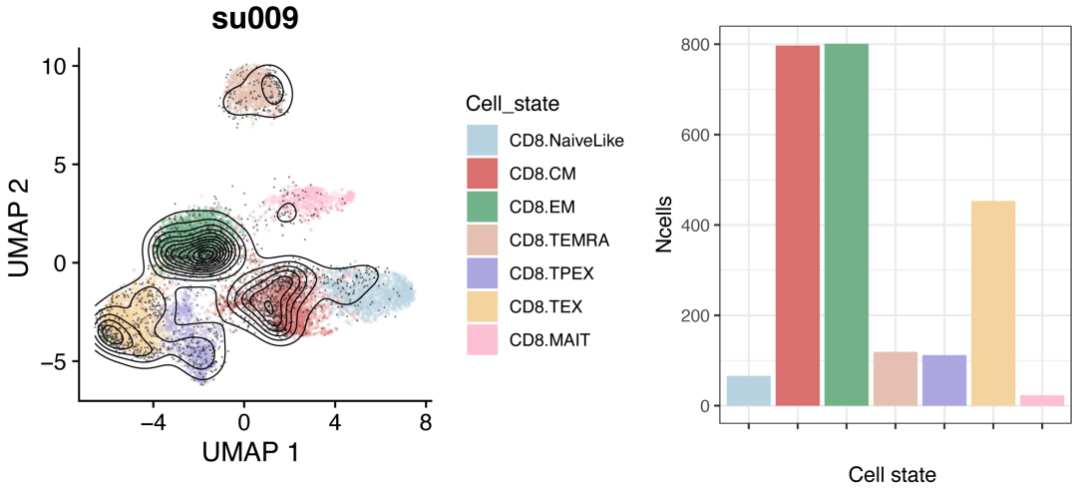
Reference embeddings and cell subtype composition for query dataset. Reference is a human CD8^+^ T-cell reference (see text); query is a representative patient (“su009”) from a basal cell carcinoma (BCC) cohort (Yost et al., 2019).
  5. Exclude small samples
    Robust analyses require a minimum number of cells in each sample. After projection and annotation, remove all samples with a small number of cells (e.g., 100 cells):
    sizes <- as.vector(lapply(seurat.projected, ncol))
    keep <- names(sizes)[sizes > 100]
    seurat.projected <- seurat.projected[keep]
    For large enough samples, we can compare their composition in terms of cell subtypes (Figure 4):
    plots <- lapply(names(seurat.projected), function(x) {
    plot.statepred.composition(ref.cd8, query = seurat.projected[[x]],
    metric = "Percent") + ggtitle(x)
    })
    wrap_plots(plots, ncol=4)
  6. Merge list of objects to obtain a single object
    For some analyses (including clonal analysis detailed below), it is useful to merge individual objects/samples (projected by patient) into a single object:
    merged.projected <- Reduce(merge.Seurat.embeddings, seurat.projected)
    Idents(merged.projected) <- "functional.cluster"
    **Figure 4.**
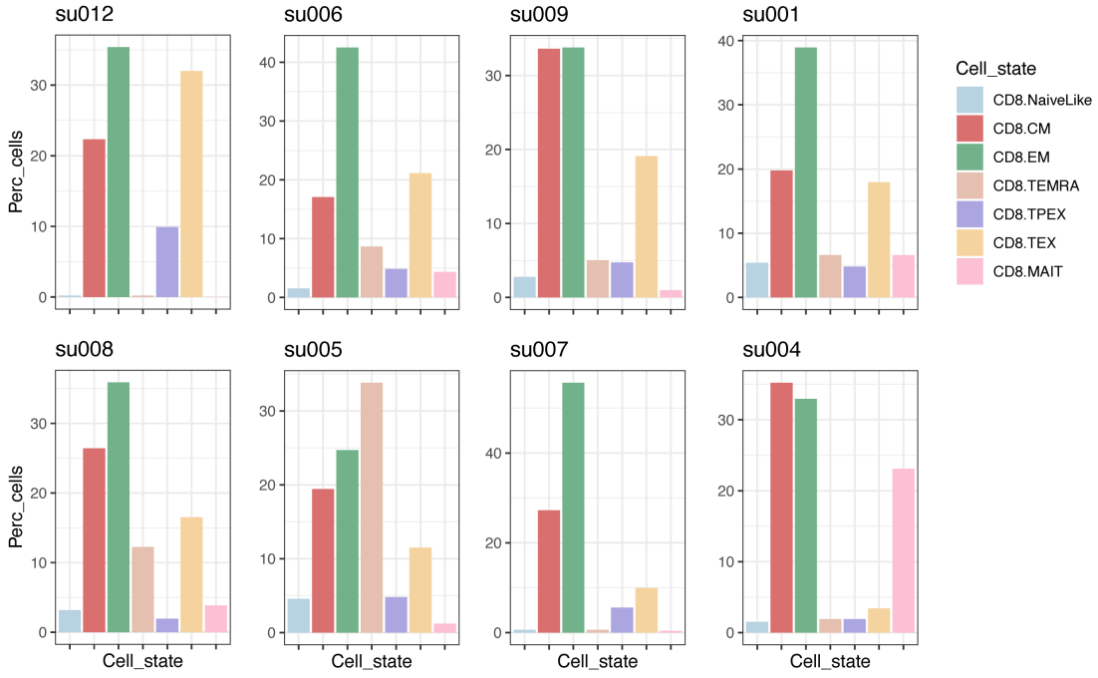
CD8^+^ T-cell subtype composition in individual tumor biopsies with at least 100 CD8^+^ T cells. Data are from basal cell carcinoma (BCC) tumor biopsies (Yost et al., 2019); plots are sorted by the fraction of CD8.TEX cells.
- **Clonal analysis**
  If the TCR information was loaded into the query Seurat object as outlined in section A, it will be available as metadata for the projected object. This allows linking the transcriptomics state to clonal information. A few examples of analyses are detailed below.
  1. Identify the most expanded clones
    Calculate the frequency of unique TCR chains per patient (e.g., as stored in “cdr3s_pat” metadata, see Note 1) to identify the most expanded clones per patient:
    freqs <- lapply(seurat.projected, function(x) {
    table(x$cdr3s_pat) / sum(!is.na(x$cdr3s_pat))
    })
    freqs <- Reduce(c, freqs)
    sorted <- sort(freqs, decreasing = TRUE)
    largest.clones <- head(sorted, 6)
  2. Locate expanded clones on the reference low-dimensional space
    TCR chains can be used to subset clones of interest (e.g., the largest clones as identified above) and inspect their distribution on the reference UMAP space (Figure 5):
    plots <- list()
    for (i in 1:length(largest.clones)) {
    ctype <- names(largest.clones)[i]
    cells <- which(merged.projected[["cdr3s_pat"]]==ctype)
    plots[[i]] <- plot.projection(ref.cd8, merged.projected[,cells])
    }
    wrap_plots(plots, ncol = 3)
    **Figure 5.**
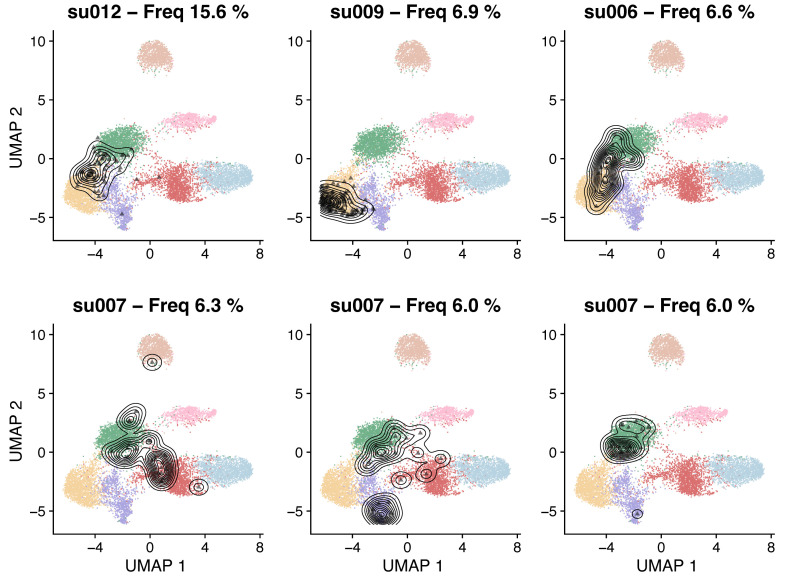
Reference UMAP embeddings highlighting with contour lines the location of the six most expanded CD8^+^ T-cell clones in basal cell carcinoma (BCC) tumor biopsies (Yost et al., 2019)
  3. Clonal expansion by T-cell subtype
    scRepertoire implements several useful functions to visualize clonal expansion and clonal diversity. Plot the number of cells in different categories of expansions, from “Single” clones to large clones (here >50 cells), by T-cell subtype (Figure 6):
    occupiedscRepertoire(merged.projected, x.axis = "functional.cluster")
    **Figure 6.**
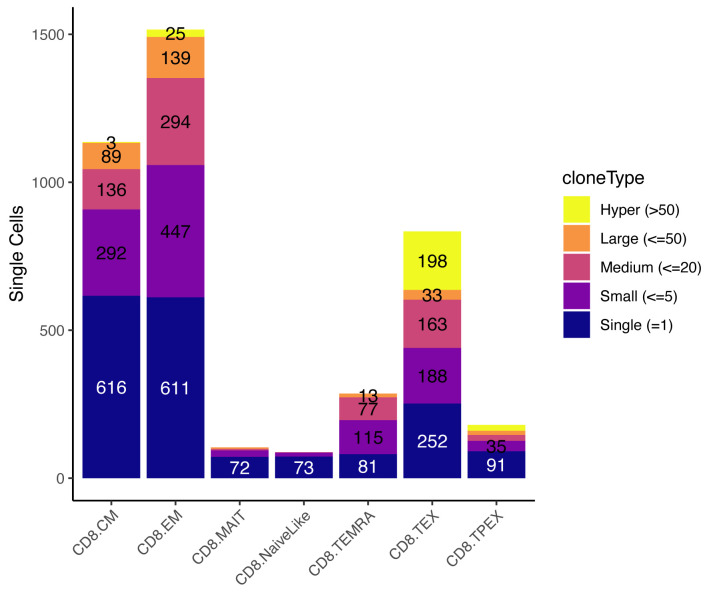
Occupied single-cell repertoire by cell subtype. The y-axis indicates the absolute number of cells, and colors identify the type of clone based on the number of cells it contains. Data from Yost et al. (2019).
  4. Clonotype proliferation rate
    High proliferation rate of a specific clonotype may indicate that the T cells with shared specificity are actively recognizing antigens in situ. We can measure proliferation at the clonal level by calculating how many cells of a clone are cycling, according to transcriptomics readouts. ProjecTILs automatically calculates cell cycling signature scores using UCell (Andreatta and Carmona, 2021). These signature scores can be used to define cell cycle stage and proliferative status (Figure 7):
    merged.projected$is.cycling <- ifelse((merged.projected$cycling.score.G1_S > 0.1 |
    merged.projected$cycling.score.G2_M > 0.1),
    yes = "Proliferating",
    no = "Resting")
    #Only consider expanded clones
    clonotypes <- table(merged.projected$cdr3s_pat)
    expanded <- names(clonotypes)[clonotypes>=2]
    frequency.proliferating <- sapply(expanded, function(x) {
    sub <- subset(merged.projected[[]], subset=cdr3s_pat == x)
    sum(sub$is.cycling == "Proliferating") / ncol(sub) })
    **Figure 7.**
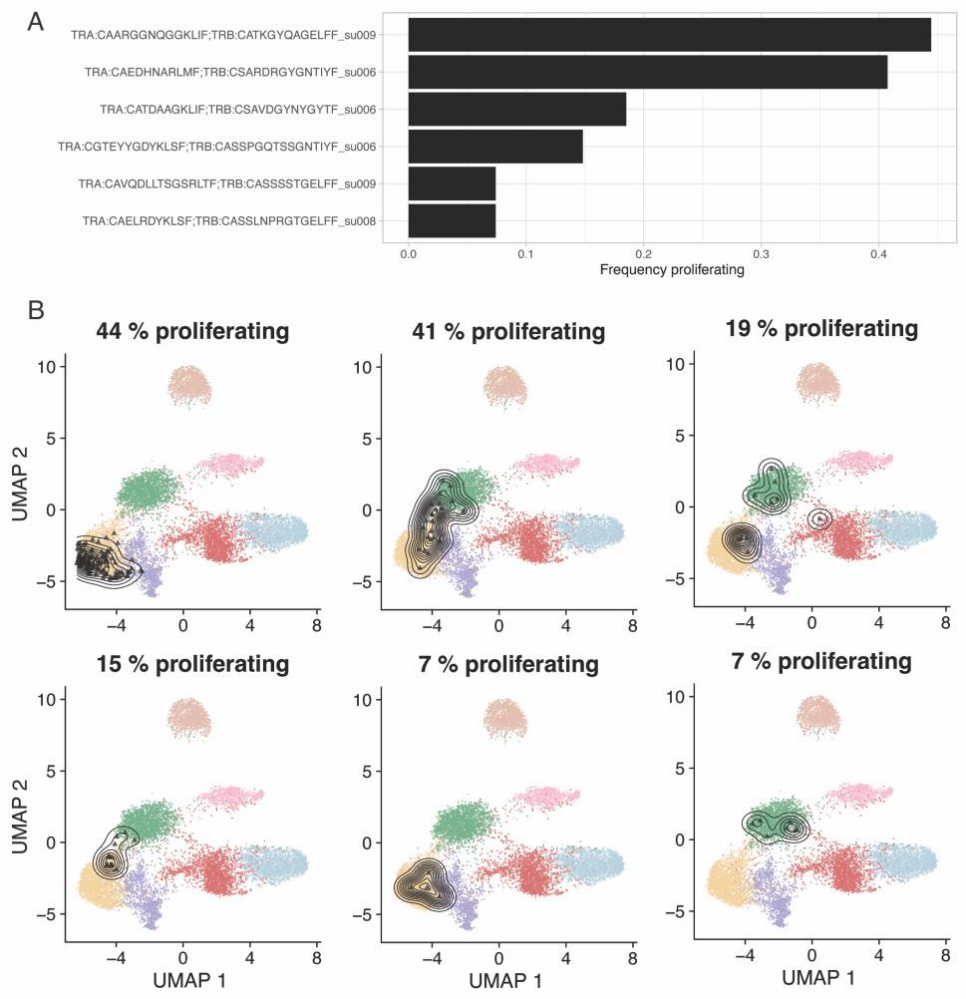
Most proliferative clones in pre-treatment biopsies from a basal cell carcinoma (BCC) cohort (Yost et al., 2019). A. Fraction of proliferating cells for the six most proliferative clones. B. Reference-embedding for the same six clones. Proliferating cells are calculated based on signature scoring of the cell cycling signatures defined by Tirosh et al. (2016).
    *Note 3: The user may want to use different gene signatures than those automatically applied by ProjecTILs, to quantify activity of additional gene programs. We refer to the UCell online documentation for interacting with Seurat objects and for custom gene signature scoring:* *https://bioconductor.org/packages/release/bioc/vignettes/UCell/inst/doc/UCell_Seurat.html*.
  5. Clonal sharing between T-cell subtypes
    Metrics of clonal overlap [e.g., Horn-Morisita index (Horn, 1966)] can be used to assess clonal sharing between samples and between T-cell subtypes. Here, we analyze the clonal sharing between subtypes (Figure 8A):
    clonalOverlap(combined, cloneCall = "cdr3s_pat", method = "morisita")
    Several additional representations of clonal overlap are available in scRepertoire, for example as circos plots (Gu et al., 2014) (Figure 8B):
    circles <- getCirclize(merged.projected,
    cloneCall = "cdr3s_pat",
    group.by = "functional.cluster")
    circlize::chordDiagram(circles)
    **Figure 8.**
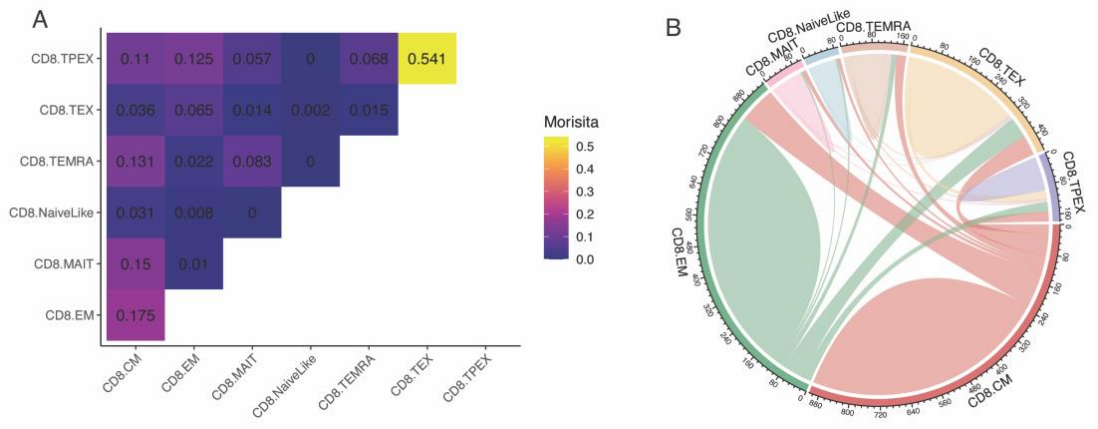
Clonal overlap between CD8^+^ T-cell subtypes. A. Morisita index for T-cell receptors (TCR) sharing between CD8^+^ T-cell subtypes. B. Circos plot visualization of clonal sharing between subtypes. Data from Yost et al. (2019).
    *Note 4: Cell type/state classification algorithms are not perfect, and there is generally some uncertainty in the predicted subtypes, especially among closely related subtypes (e.g., NaiveLike and CM/Central Memory). Moreover, some cells might display intermediate states of differentiation, transitioning from one state into another. These factors might lead to some* background noise *for TCR sharing/Morisita index between transcriptionally related cell states (e.g., in Figure 8, a Morisita index of 0.031 between NaiveLike and CM is very unlikely to be meaningful). It is strongly advised to analyze multiple independent samples to support hypotheses of TCR sharing between groups.*
  6. Clonotype bias towards specific cell states
    In certain settings, it may be of interest to identify clones that are significantly composed of T cells of a certain subtype. We have previously devised a metric to measure clonotype bias and applied it to investigate if virus-specific naïve CD4^+^ T-cell clones were preferentially differentiating into a specific effector state, or whether multiple differentiation fates were equally likely (Andreatta et al., 2022). scRepertoire implements a function to calculate clonotype bias (Figure 9A):
    clonotypeBias(merged.projected, cloneCall = "cdr3s_pat", split.by = "patient",
    group.by = "functional.cluster", min.expand = 10)
    **Figure 9.**
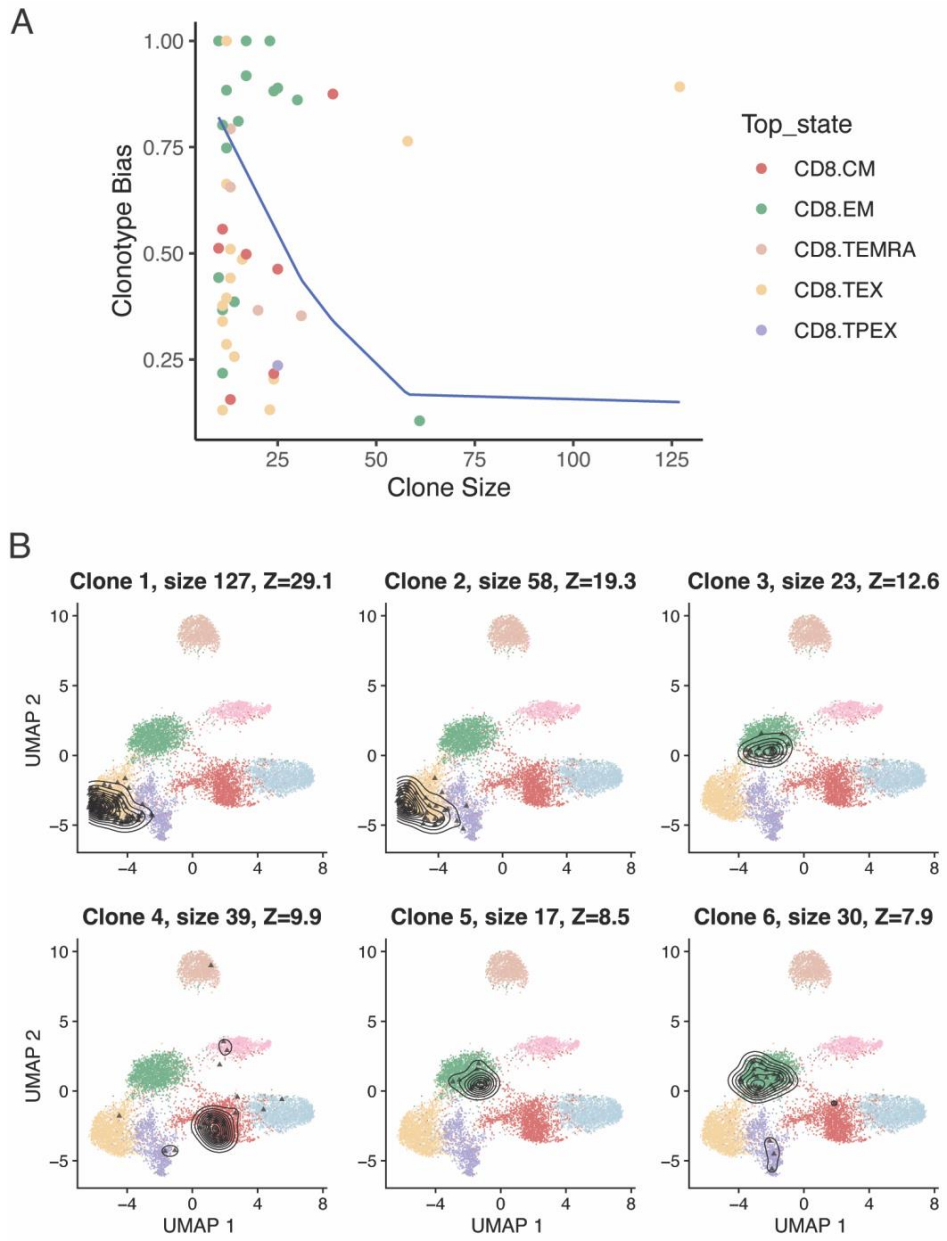
Clonotype bias towards specific cell states. A. Clonotype bias as a function of clonal size, for expanded CD8^+^ T-cell clones in basal cell carcinoma (BCC) tumors (Yost et al., 2019). The blue line approximates the upper bound of a 95% confidence interval of the expected clonotype bias distribution (i.e., the clonotype bias expected by chance if there was no biological association between clonotype and cell state). B. T-cell clones with most significant clonotype bias, ranked by Z-score.
    The same function can be used to return a table, by setting *exportTable=TRUE*, from which we can extract the most significantly biased clones according to their Z-score (Figure 9B):
    biased <- clonotypeBias(merged.projected, cloneCall = "cdr3s_pat", split.by = "patient",
    group.by = "functional.cluster", min.expand = 5, exportTable=TRUE)
    most.biased <- biased[order(biased$Z.score, decreasing = TRUE),]
    plots <- list()
    for (i in 1:6) {
    ctype <- most.biased[i, "Clone"]
    cells <- which(merged.projected [["cdr3s_pat"]]==ctype)
    title <- sprintf("Clone %s - size %s - %s", i, size, patient)
    plots[[i]] <- plot.projection(ref.cd8, merged.projected [,cells])
    }
    wrap_plots(plots, ncol = 3)

## Data analysis

Fully reproducible R code that generates the results and figures in this protocol, including all pre-processing steps, is available on GitHub: https://github.com/carmonalab/Tcell_clonal_analysis. A comprehensive vignette with more information on scRepertoire and its functions can be found at:

https://ncborcherding.github.io/vignettes/vignette.html. Several case studies of applications of ProjecTILs for reference-based analysis of single-cell data are available at: https://carmonalab.github.io/ProjecTILs_CaseStudies.

## General notes and troubleshooting

Commercially available single-cell RNA-sequencing technologies have opened the opportunity to study the association of T-cell states and clonality at large scale. However, scRNA-seq experiments typically produce less than 10,000 high-quality single-cell transcriptomes per sample. Depending on the tissue analyzed, and whether or not T cells have been specifically purified, the number of sequenced T cells obtained, even from inflamed tissues, can be very low. As a result, only a small fraction of the complete TCR repertoire is typically sampled. Under-sampling leads to inaccurate estimations of clonal diversity (e.g., Shannon entropy). For this reason, in this protocol we suggest to exclude from analysis samples with very few cells and we avoided the use of clonal diversity metrics, such as Shannon entropy, Gini-Simpson index, and Gini coefficient, that are particularly sensitive to under-sampling (Chiffelle et al., 2020). Instead, we focused the analysis on the largest clonotypes in each sample. Clonal sharing between samples (e.g., Morisita index) is also affected by the low number of observations. Thus, clonal diversity and clonal sharing metrics should be interpreted with caution, and importantly, confirmed in independent samples.

**Troubleshooting**

Download of large objects in R (as in the case of single-cell datasets and reference maps) may occasionally fail due to connection timeout. This commonly manifests in errors such as “object X is invalid.” Try increasing download timeout using the following command within the R session:

options(timeout = max(900, getOption("timeout")))
